# *Drosophila mojavensis* – *chico*

**DOI:** 10.17912/micropub.biology.000677

**Published:** 2022-11-15

**Authors:** Hannah Congleton, Cole A. Kiser, Patricia A. Colom Diaz, Elizabeth Schlichting, Dorothy A. Walton, Lindsey J. Long, Laura K. Reed, Juan Carlos Martinez-Cruzado, Chinmay P. Rele

**Affiliations:** 1 Oklahoma Christian University, Edmond, OK USA; 2 The University of Alabama, Tuscaloosa, AL USA; 3 University of Puerto Rico at Mayagüez, Mayaguez, PR Puerto Rico

**Figure 1.**
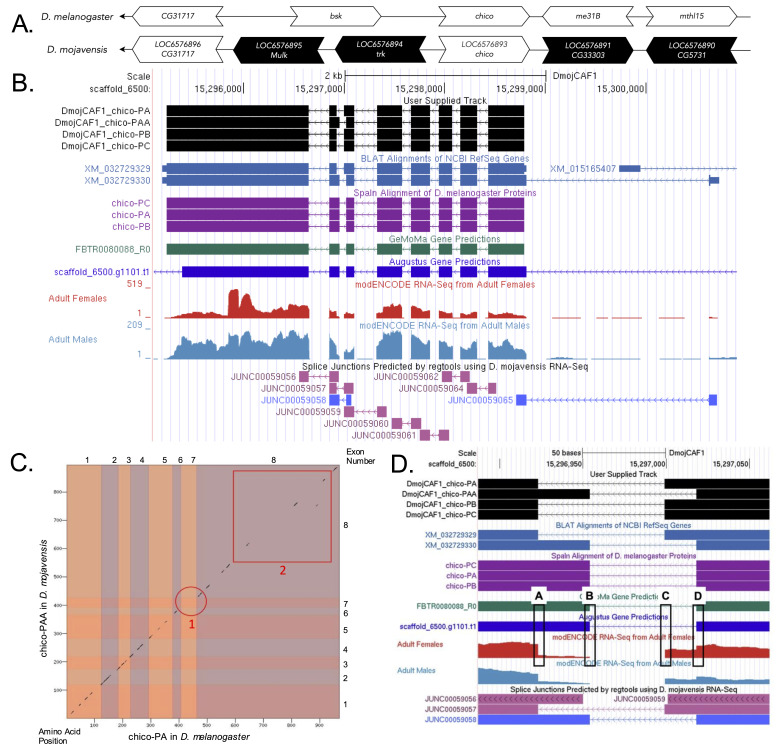
**
(A) Synteny comparison of the genomic neighborhoods for *chico* in *Drosophila melanogaster* and *D. mojavensis* .** Thin underlying arrows indicate the DNA strand within which the target gene– *chico* –is located on the negative strand in *D. melanogaster* (top) and *D. mojavensis* (bottom). The wide gene arrows pointing in the same direction as *chico* are on the same strand relative to the thin underlying arrows, while wide gene arrows pointing in the opposite direction of *chico* are on the opposite strand relative to the thin underlying arrows. White gene arrows in *D. mojavensis* indicate orthology to the corresponding gene in *D. melanogaster* , while black gene arrows indicate non-orthology. Gene symbols given in the *D. mojavensis* gene arrows indicate the orthologous gene in *D. melanogaster* , while the locus identifiers are specific to *D. mojavensis* . **(B) Gene Model in GEP UCSC Track Data Hub (Raney *et al* ., 2014).** The coding-regions of *chico* in *D. mojavensis* are displayed in the User Supplied Track (black); coding exons are depicted by thick rectangles and introns by thin lines with arrows indicating the direction of transcription. Subsequent evidence tracks include BLAT Alignments of NCBI RefSeq Genes (dark blue, alignment of Ref-Seq genes for *D. mojavensis* ), Spaln of D. melanogaster Proteins (purple, alignment of Ref-Seq proteins from *D. melanogaster* ), Transcripts and Coding Regions Predicted by TransDecoder (dark green), RNA-Seq from Adult Females and Adult Males (red and light blue, respectively; alignment of Illumina RNA-Seq reads from *D. mojavensis* ), and Splice Junctions Predicted by regtools using *D. mojavensis* RNA-Seq (Chen *et al.* , 2014; SRP006203). Splice junctions shown have a read-depth of 10-49 and 100-499 supporting reads in blue and pink. **(C) Dot Plot of chico-PA in *D. melanogaster* ( *x* -axis) vs. the new PAA isoform in *D. mojavensis* ( *y* -axis).** Amino acid number is indicated along the left and bottom; coding-exon number is indicated along the top and right, and exons are also highlighted with alternating colors. The red circle labeled 1, Regions which indicate a lack of similarity between the two sequences are highlighted in red (Region 1; Region 2). **(D) Gene model in GEP UCSC displaying RNA-Seq and Splice Junctions data of exons six and seven of the ortholog in *D. mojavensis* .** Boxes A and B indicate RNA-Seq drop-offs, boxes C and D indicate their corresponding splice acceptors and the region in between the boxes represent introns. The increase of RNA-Seq data in exons 6 and 7 and the existence of splice junction JUNC00059058 support that an additional isoform is present in *D. mojavensis* (chico-PAA).

## Description

***Introduction*** The insulin signaling pathway is a highly conserved pathway in animals and is central to nutrient uptake (Hietakangas and Cohen 2009; Grewal 2009). A key component of the pathway, encoding a substrate of the Insulin Receptor (InR) protein, *chico* binds to the InR and thus is also referred to as the Insulin Receptor Substrate (IRS). The IRS *chico* is essential to the control of cell and organ size (Goderdhan et al. 1999; Bohni et al. 1999), overall growth (Bakopoulos et al. 2020), including the sex-biased control of cell size and growth (Millington et al. 2021; Kim and O’Connor 2021), and is involved in lifespan (Clancy et al. 2001). We propose a gene model for the *D. mojavensis* ortholog of the *D. melanogaster* chico *(chico)* gene. The genomic region of the ortholog corresponds to the uncharacterized protein LOC6576893 (RefSeq accession XP_032585220.1) in the dmoj_caf1 Genome Assembly of *D. mojavensis* (GenBank Accession: GCA_000005175.1 - Chen *et al.* , 2014; SRP006203). This model is based on RNA-Seq data from *D. mojavensis* (Chen *et al.,* 2014; SRP006203) and *chico* in *D. melanogaster* using FlyBase release FB2022_04 (GCA_000001215.4; Larkin *et al.* , 2021). *D.* *mojavensis* is part of the *mulleri complex* in the *repleta* species group within the subgenus *Drosophila* of the genus *Drosophila* (Wasserman 1992, Durando et al, 2000 *).* It was first described by Patterson (Patterson and Crow, 1940). *D. mojavensis* specializes on rotting cactus as its host and is found in the Mojave and Sonoran Deserts of the southwestern United States and northwestern Mexico including the Baja Peninsula, as well as on the channel-islands off the coast of California (https://www.taxodros.uzh.ch). The Genomics Education Partnership maintains a mirror of the UCSC Genome Browser (Kent WJ *et al.* , 2002; Gonzalez *et al.* , 2021), which is available at https://gander.wustl.edu .

***Synteny***

The target gene, *chico,* occurs on chromosome 2L in *D. melanogaster* and is flanked upstream by *CG31717* and basket *(bsk)* and downstream by maternal expression at 31B *(me31B)* and methuselah-like 15 *(mthl15)* . The *tblastn* search of *D. melanogaster* chico-PA (query) against the *D. mojavensis* (GenBank Accession: GCA_000005175.1) Genome Assembly (database) placed the putative ortholog of *chico* within scaffold scaffold_6500 (CH933807.1) at locus LOC6576893 (XP_032585220.1)— with an E-value of 0.0 and a percent identity of 44.97%. Furthermore, the putative ortholog is flanked upstream by LOC6576896 (XP_015020894.1) LOC6576895 (XP_002002875.4) and LOC6576894 (XP_015020893.1), which correspond to *CG31717,* Multi-substrate lipid kinase *(Mulk)* and trunk *(trk)* in *D. melanogaster* (E-value: 2e-77, 3e-155 and 3e-100; identity: 65.64%, 54.44% and 65.58%, respectively, as determined by *blastp* ; Figure 1A, Altschul *et al.* , 1990). The putative ortholog of *chico* is flanked downstream by LOC6576891 (XP_002002871.1) and LOC6576890 (XP_002002870.1), which correspond to *CG33303* and *CG5731* in *D. melanogaster* (E-value: 0.0 and 0.0; identity: 65.78% and 84.56%, respectively, as determined by *blastp* ). The putative ortholog assignment for *chico* in *D. mojavensis* is supported by the following evidence: Although the genes surrounding the *chico* ortholog are not orthologous to the genes at the same locus in *D. melanogaster* , we conclude that LOC6576893 is the correct ortholog of *chico* in *D. mojavensis* (Figure 1A), supported by strong e-values and percent identities and the lack of other matches while performing the *blastp* search.

***Protein Model***

Consistent with the *blastp* search result which shows 44.97% identity between *D. melanogaster* chico-PA and the *D. mojavensis* gene model as well as the low sensitivity parameters used to generate the dot plot (i.e., word size = 3; neighborhood threshold = 11), the dot plot of the two protein sequences contain multiple large gaps along the diagonal. *chico* in *D. mojavensis* has 3 identical protein-coding isoforms (chico-PA, chico-PB and chico-PC; Figure 1B). Isoform (chico-PA, chico-PB, chico-PC) contains 8 protein-coding exons. Relative to the ortholog in *D. melanogaster* , the coding-exon number is conserved. The sequence of chico-PA in *D. mojavensis* has 44.97% identity (E-value: 0.0) with the protein-coding isoform chico-PA in *D. melanogaster* , as determined by *blastp* (Figure 1C). While conducting our research, the presence of a new isoform of chico in *D. mojavensis* was discovered. The isoform identified as PA *D. mojavensis* is orthologous to the PA isoform in *D. melanogaster* and the PAA isoform is the novel isoform. Lower score Splice Junctions data due to lower levels of RNA-Seq data indicate the chico-PAA isoform is expressed at lower levels, or in fewer cells, than the chico-PA isoform (Figure 1D). Coordinates of this curated gene model are stored by NCBI at GenBank/BankIt (accession BK059533). These data are also archived in the CaltechDATA repository (see “Extended Data” section below).

***Special characteristics of the protein model***

**Novel isoform:** A new isoform of chico (chico-PAA) has been discovered based on the available RNA-Seq data and Splice Junctions data (Figure 1D). RNA-Seq data drops off in two regions, labeled A and B, and RNA-Seq data increase are displayed in two regions, labeled C and D. Blue and pink data tracks beneath the RNA-Seq data represent Splice Junctions, with pink splice junctions having a higher score than blue splice junctions (minimum read-depth scores found in above image caption of Figure 1A). The existence of splice junction JUNC00059058 as well as the decrease and sharp increase in RNA-Seq data in exons 6 and 7 which overlap the exons of isoform PA indicates an additional isoform (Figure 1D).

## Extended Data

Description: FASTA. Resource Type: Model. DOI: 10.22002/n7ehe-xej65

Description: GFF. Resource Type: Model. DOI: 10.22002/26d2v-g8r63

Description: Peptide Sequence. Resource Type: Model. DOI: 10.22002/vbfq6-4z544
